# Biomarkers of peripheral muscle fatigue during exercise

**DOI:** 10.1186/1471-2474-13-218

**Published:** 2012-11-08

**Authors:** Josef Finsterer

**Affiliations:** 1 Krankenanstalt Rudolfstiftung, Postfach 20, 1180, Vienna, Austria

**Keywords:** Biomarker, Biological marker, Muscle fatigue, Monitoring, Muscle exercise, Exercise fatigue

## Abstract

**Background:**

Biomarkers of peripheral muscle fatigue (BPMFs) are used to offer insights into mechanisms of exhaustion during exercise in order to detect abnormal fatigue or to detect defective metabolic pathways. This review aims at describing recent advances and future perspectives concerning the most important biomarkers of muscle fatigue during exercise.

**Results:**

BPMFs are classified according to the mechanism of fatigue related to adenosine-triphosphate-metabolism, acidosis, or oxidative-metabolism. Muscle fatigue is also related to an immunological response. impaired calcium handling, disturbances in bioenergetic pathways, and genetic responses. The immunological and genetic response may make the muscle susceptible to fatigue but may not directly cause muscle fatigue. Production of BPMFs is predominantly dependent on the type of exercise. BPMFs need to change as a function of the process being monitored, be stable without appreciable diurnal variations, correlate well with exercise intensity, and be present in detectable amounts in easily accessible biological fluids. The most well-known BPMFs are serum lactate and interleukin-6. The most widely applied clinical application is screening for defective oxidative metabolism in mitochondrial disorders by means of the lactate stress test. The clinical relevance of most other BPMFs, however, is under debate, since they often depend on age, gender, physical fitness, the energy supply during exercise, the type of exercise needed to produce the BPMF, and whether healthy or diseased subjects are investigated.

**Conclusions:**

Though the role of BPMFs during fatigue is poorly understood, measuring BPMFs under specific, standardised conditions appears to be helpful for assessing biological states or processes during exercise and fatigue.

## Review

### Introduction

Fatigue in response to exercise (exercise (-induced) fatigue) can be caused by mental disorders, organic central nervous system (CNS) abnormalities (central fatigue), or by peripheral nervous system (PNS) dysfunction or skeletal muscle disease (peripheral, muscle, contractile, or mechanical fatigue, contractile impairment, loss of force generating capacity) [1,2]. Factors that contribute to feeling tired include neurological and non-neurological causes [3].

Neurological causes include:

Central fatigue

Mental disorders

Organic CNS abnormalities

Peripheral fatigue

PNS (neural adaptations)

Skeletal muscle diseases (contractile fatigue, contractile impairment)

Non-neurological causes include:

Cardiac disorders

Pulmonary disorders (e.g. chronic obstructive pulmonary disease (COPD)

Hematologic disorders

Metabolic diseases

Renal problems

Malignancies

Overtraining syndrome

Chronic fatigue syndrome (CFS)

Exercise-induced muscle fatigue has to be clearly delineated from fatigability in the resting stage (fatigue prior to exercise), which may be caused by similar or other factors than exercise-induced muscle fatigue. Exercise-induced muscle fatigue is defined as a reversible loss of muscle force (muscle contractility) during work over time (peripheral muscle fatigue during exercise) [4,5]. Clinically useful biomarkers are available to quantify the degree and course of muscle fatigue during exercise and disease. This review is directed towards researchers and clinicians and aims to describe recent advances and future perspectives concerning the most important biomarkers of muscle fatigue during exercise.

### Exercise

Voluntary muscle exercise can be carried out in an incremental or constant manner (endurance), or in a dynamic (cycling, running) or static mode. Exercise can also be exhaustive or non-exhaustive. Exhaustive exercise includes acute, very prolonged exercise, a period of intensified training, non-functional overreaching and the overtraining syndrome. Exercise can be carried out under aerobic or anaerobic conditions. Examples of partially anaerobic exercise include sprinting, where maximal efforts last <1 minute, and exercise under high-force isometric conditions. Only a very few types of exercise are entirely anaerobic, and these necessarily last for only a few seconds. Contractions during exercise may be isometric or non-isometric, isokinetic (constant velocity throughout movement) or non-isokinetic (changing velocity during contraction), or concentric (muscle shortening) or eccentric (muscle lengthening). All of these different conditions are not exclusive, i.e. a cycling exercise may be constant, dynamic, non-isometric and concentric.

### Exercise-induced muscle fatigue, fatigability, tiredness, and recovery

Muscle fatigue is defined as decreased ability to generate appropriate amounts of muscle force or power during on-going contractile activity. Muscle fatigue may vary according to the cause or underlying mechanisms, with variable rates of recovery. For example, fatigue brought on by a marathon is different from fatigue that prevents a 10th bench press repetition at a given resistance. Exercise-induced muscle fatigue can occur shortly after onset of exercise (acute muscle fatigue) or after a constant, high-intensity exercise had been carried out for a prolonged period of time (delayed exercise-induced fatigue), which is characterized by tiredness only after a longer duration of constant exercise. Acute muscle fatigue is usually maximal immediately post-exercise. Exercise-induced muscle fatigue can develop under a sub-maximal as well as maximal intensity exercise. Exercise-induced fatigue can occur in healthy or diseased subjects, and depends on age, gender, physical fitness, mode and duration of exercise, and the underlying disease.

Contrary to fatigue, the term fatigability is defined as rate of loss of muscle force over time. Fatigability is an interactive determinant of the state of fatigue versus rested. Only few studies have addressed the relation between fatigability and biomarkers of muscle fatigue. Muscle fatigue has to be delineated from the sensation of tiredness. Muscle fatigue that develops during submaximal aerobic exercise is not necessarily reflected by a sensation of tiredness, but a reduced ability to develop force and power. Recovery from fatigue is defined as the rate at which muscle function returns to baseline after becoming fatigued. Recovery represents an important marker of fatigue severity and biomarker responsiveness. Like fatigability, recovery represents an interactive determinant of the state of fatigue versus rested. Both, fatigue severity and recovery are influenced by fatigability and the degree to which muscle dysfunction is provoked.

### Fatigue versus muscle damage

Decrease in force production during exercise can be regarded as a safety mechanism. If fatigue would not occur or was delayed, structural damage to muscle cells and supportive tissues would occur during the workout. Skeletal muscle is generally composed of slow-twitch and fast-twitch muscle fibres [6]. Slow-twitch fibres are also referred to as type-I, whereas fast-twitch fibres are referred to as type-IIa, typeIIx, or type-IIb [6]. The differentiation is based on histochemical staining for myosin adenosine-triphosphatase (ATPase) and the type of myosin heavy chain [6]. Slow-twitch fibres are better equipped to work aerobically, whereas fast-twitch fibres are better equipped to work anaerobically. Particularly in type-II fibres, CK-activity serves as a temporal or spatial buffer of the adenosine-triphosphate (ATP) content [7]. Both type-I and type-II fibres produce interleukin (IL)-6 [8]. Muscle fatigue, which can be recovered from fairly quickly, must be clearly distinguished from muscle damage, which usually takes a longer recovery period. Since muscle fatigue is not accompanied by structural damage to myofibrils and muscle damage can occur without exercise inducing it (e.g. contusion, excessive stretching, rhabdomyolysis), biomarkers for both of these conditions must be differentiated. There are, however, conditions under which biomarkers of muscle fatigue coexist with biomarkers of muscle damage (e.g. inflammatory biomarkers). In particular, high-intensity eccentric contractions can result in muscle damage, making it difficult to determine whether the biomarker is indicative of fatigue or muscle damage.

### Biomarkers

A biomarker (biological marker) is a measurable product or substance of an organism that is used as an indicator of a biological state to objectively measure physiological or pathogenic processes in the body that occur during health, disease or in response to pharmacological treatment [9]. In medicine, a biomarker can also represent a substance that is introduced into an organism to examine organ functions or other aspects of health (e.g. single photon emission-computed tomography tracers, which are used as radioactive isotopes to assess the number of dopamine-producing cells). A biomarker can also be a substance that indicates a particular disease state (e.g. acetylcholine receptor antibodies indicate myasthenia gravis). More specifically, a biomarker indicates a change in the expression or state of a protein that correlates with the risk or progression of a disease, or with the susceptibility of the disease to a given treatment (e.g. tumour markers). Criteria a biomarker needs to meet are that it changes as a function of the process being monitored, can be accurately measured; is stable and does not show diurnal variations, correlates with exercise intensity, and is present in detectable amounts in easily accessible biological fluids [10]. Biomarkers of muscle fatigue should be selected such that they do not overlap with biomarkers of muscle damage.

### Biomarkers of peripheral muscle fatigue

Biomarkers can be categorized according to the type of exercise or workload carried out (short or long term, aerobic exercise or anaerobic exercise, or both (e.g. lymphocytes increase during aerobic exercise)) [11] or according to the delay before which they are detectable after onset or discontinuation of exercise (Table 1) [12].

**Table 1:** Criteria for the classification of biomarkers of muscle fatigue

**Criterion**	**Mode of exercise**
Type of exercise	Aerobic/anaerobic
Type of contraction	Incremental/constant
	Isometric/non-isometric
	Isokinetic/non-isokinetic
	Dynamic/static
	Concentric/eccentric

Investigated compartment	Fluids (serum, saliva, urine), cells (blood, muscle)
Type of individual	Human being, animal
Delay of production	Early production/delayed production
Metabolic state	Anabolic/catabolic
Biochemical reaction	Acidosis, immunologic, oxidative, hormonal

The concentration of a biomarker produced depends on the amount of fatigue that is induced and on the type and duration of the exercise. A further classification relies on the biochemical pathway from which the biomarker originates (Table 2).

**Table 2:** Classification of biomarkers of muscle fatigue

**Biomarker**	**Source**
ATP metabolism biomarkers	
Lactate	Serum
Ammonia	Serum
Oxipurines	Serum
Oxidative stress biomarkers	
Lipid peroxidation	
Thiobarbituric acid reactive substances (TBARS)	Serum
Isoprostanes (F2-isoP)	Serum, urine
Protein peroxidation	
Protein carbonyls	Serum
Antioxidative capacity	
Glutathione (GSH)	Serum
Glutathione peroxidase (GTX)	Serum, leukocytes
Catalase	Serum
Total antioxidant capacity (TAC)	Serum
Inflammatory biomarkers	
Leukocytes	Blood
Interleukin-6	Serum
TNF-α	Serum

However, the most plausible classification of biomarkers of muscle fatigue follows the mechanisms of fatigue and the metabolic changes during fatigue. Since there is no single cause of muscle fatigue, there is also no single biomarker for assessing muscle fatigue. Studies of several biomarkers during muscle strength decline showed that multiple elevated catabolic biomarkers are a better predictor of muscle fatigue than a single biomarker [12].

Biomarkers of muscle fatigue are different for exercise that lasts 20s in duration, which have an anaerobic energy demand of up to 90%, compared to exercise that lasts 20s to 1 minute, which is maintained by both an anaerobic and aerobic energy supply, and compared to exercise that lasts > 1 minute, for which the energy supply is > 50% aerobic. Biomarkers of muscle fatigue from low- and high-intensity exercise are thus different and their return to normal values depends on the rate of recovery of muscle performance. If fatigue is still present but the biomarker is back to pre-exercise values, then the value of such a biomarker is questionable. A simple way to assess fatigue resistance is to compare the maximal force a muscle (group) can generate after contractile activity with the force/power the muscle could generate before onset of the contractile activity [13].

### Biomarkers classified according to muscle fatigue mechanism

Initially, it was believed that acidosis is the major cause of muscle fatigue [14]. Meanwhile, however, it turned out that there is no single mechanism but multiple mechanisms of fatigue, of which the most important include: 1. acidosis and depletion of ATP due to increased consumption or decreased provision, reflected by the biomarkers serum lactate, ammonia, and oxipurines, and 2. overproduction of reactive oxygen species (ROS), reflected by lipid peroxidation biomarkers, protein peroxidation biomarkers, and antioxidantive capacity biomarkers. Less well-defined causes of muscle fatigue include local inflammatory reactions reflected by the biomarkers leukocytes, tumour necrosis factor (TNF)-α, and ILs, altered Ca^2+^ release and handling [2], disturbed bioenergetic pathways (e.g. glycogen depletion), or the impaired endocrine function of muscle cells. Muscle fatigue is also reflected by an increase in the activity of genes involved in any of these mechanisms. Local inflammatory reactions to exertion during workload, which continue during the hours after exercise, may not only reflect muscle fatigue but also damage that occurred during exercise.

#### ATP metabolism

To which degree shortage in ATP contributes to muscle fatigue is under debate. Though decrease of muscle ATP can be observed during repetitive or prolonged muscle contraction, there are indications that decrease in high energy compounds (e.g. ATP, phospho-creatine (PCr)) are not enough to result in even a small level of muscle dysfunction [15]. There are even observations, indicating that ATP may remain constant with decreasing force [16]. However, there are also indications that decrease of ATP at specific locations or in association with the function of transport systems may contribute to the development of muscle fatigue. An argument against ATP-depletion as a fatigue mechanism is that the muscle is surprisingly tolerant to an increase in ADP and to a decrease in energy from ATP [17]. Generally, ATP is synthesized by four processes: the conversion of PCr at onset of exercise, anaerobic glycolysis during intensive short-term muscle work, the conversion of two ADP molecules to ATP and AMP during heavy exercise, and aerobic glycolysis under most exercise conditions [18].

At the beginning of exercise the total adenine nucleotide pool (ATP + ADP + adenosine-monophosphate (AMP)) remains constant, since ADP is reconverted to ATP in order to maintain the delivery of energy for exercise [18]. With increasing duration and intensity of exercise, the ATP:adenosine-diphosphate (ADP) ratio decreases due to excessive ATP consumption (Gibb’s free energy of ATP hydrolysis declines) and the total adenine nucleotide pool decreases due to AMP deamination. Progressive accumulation of AMP deamination products, which include inosin-monophosphate (IMP) and ammonia [18], causes fatigue but there is only a trivial correlation between fatigue and IMP concentration. Patients with AMP deaminase deficiency are more susceptible to fatigue than controls, suggesting that this enzyme plays a key role in fatigue resistance [18]. Fatigue can also result from a lack of PCr and an accumulation of intracellular phosphorus with decreasing force [19]. Since the resynthesis of PCr and the removal of phosphorous are both oxygen dependent, there might be a link between aerobic fitness and fatigue during work [19].

A key role for muscle fatigue plays intracellular acidosis. Even minimal decrease in muscle pH interferes with cross-bridge binding and ATPase activity due to competitive binding and reduced enzyme function. Cross bridge detachment is unaffected by fatigue whereas cross bridge attachment is reduced during fatigue [16]. Decreased intracellular pH may additionally impair oxidative enzyme activity and may adversely affect ryanodine receptor function [20]. Although some studies showed a relation between exercise and biomarkers of ATP-metabolism, there are still concerns on how to assess fatigue by measuring them. The most well-known biomarkers of muscle fatigue deriving from ATP metabolism include serum lactate, serum ammonia and the oxipurines hypoxanthine and xanthine.

##### Lactate

In cases, in which the oxidative phosphorylation of ADP to generate ATP fails to meet the energy requirement of the myocyte, which is the case under acidic conditions, the production of ATP shifts from aerobic processes (the processing of glucose/glycogen, lipids or amino acids) to anaerobic glycolysis or glycogenolysis (the processing of glucose/glycogen) [4]. Whether the change to anaerobic glycolysis results in lactate production only in case of inadequate oxygen supply to allow the transport of pyruvate to mitochondria, is under debate. Generally, elevated serum lactate reflects that the aerobic ATP generation is insufficient for the required ATP generation and needs to be supplemented with anaerobic ATP generation. The percentage of the maximal workload during increasing exercise at which lactate surmounts above normal levels is called the lactate threshold. World-class athletes have a lactate threshold of 70-90% compared to 50-60% in untrained individuals. Lactate production is enhanced when the level of high-energy phosphates other than ATP (e.g. PCr) is reduced, as determined by phosphorus magnetic resonance (MR) spectroscopy [4]. Lactic acidosis also results from liver dysfunction since lactate is transferred into glucose via the Cori cycle in the liver. If lactate clearance via the Cori cycle no longer keeps up with lactate production, lactic acidosis is the consequence. Meanwhile, however, there are also indications that the muscle not only plays an important role in lactate production but also in lactate clearance [21].

Reference limits for serum lactate are reported as > 2 mmol/l [22]. Serum lactate does not increase with age, does not differ between the genders, and does not seem to depend on the physical fitness of the proband but increases with the intensity of exercise in healthy and diseased subjects and in trained and untrained individuals when exercise exceeds the lactate threshold [23]. With incremental exercise, serum lactate increases exponentially above a certain work-rate. When fatigue was compared between male adolescents and adults, post-exercise serum lactate increased more in adults as compared to adolescents [24]. In prolonged constant-load endurance exercise, lactate typically increased by < 5 mmol/l. Lactate also increased during and after exercise in animals [25]. Generally, lactate increases at lower relative workloads in patients as compared to healthy subjects. Lactate increases at low workloads particularly in patients with impaired oxidative phosphorylation (mitochondrial disorders) and patients with pulmonary disease. In patients with COPD, serum lactate was found to increase up to the maximal capacity during a stepwise exercise test on an ergocycle [26]. Lactic acidosis can result in hypocalcaemia and hypomagnesaemia [23]. Despite these influences on serum lactate levels, lactate appears to be a promising biomarker of muscle fatigue if workload conditions are standardised [4].

##### Ammonia

If the consumption of ATP exceeds the ATP supply, the ATP:ADP ratio decreases and impairs the functioning of the major ATPases in myocytes, including myosin ATPase (70%), endoplasmatic reticulum Ca-ATPase (25%), and sarcolemmal Na/K-ATPase [4]. Impairment of myocyte ATPases leads to fatigue [4]. In order to maintain the ATP: ADP ratio, the enzyme adenylate-kinase transfers one energy-rich phosphate group from one ADP to another ADP, resulting in one ATP and one AMP. AMP is subsequently degraded by the enzyme AMP-deaminase to IMP and ammonia [27]. AMP acts by activating the AMP-kinase (AMPK), which is the key sensor of cellular energy stress [28]. The AMPK in myocytes is activated by exercise and is partially responsible for the acute metabolic response of the muscle to exercise [29]. It also improves the substrate supply for metabolic pathways producing ATP, and inhibits anabolic processes to spare ATP [28]. AMPK is particularly involved in the switch from the anaerobic metabolism of glycogen to the oxidative metabolism of blood glucose and fatty acids [29]. AMPK is also responsible for long-term metabolic adaptations, such as the increase in number of mitochondria to aerobic exercise in endurance training [29]. Ammonia is usually determined in the serum.

Reference limits of ammonia have been repeatedly reported but depend on a number of variables. Serum ammonia does not seem to be age-dependent [30]. Dependency on sex, and physical fitness was not extensively investigated, but increase of ammonia after sprint exercise was higher in men as compared to females [31]. In trained and untrained healthy subjects performing a maximal ergocycle exercise, ammonia increased by 32% during exercise. Because AMP degradation is enhanced during exercise, serum ammonia and intracellular IMP levels concomitantly increase [4,32]. Since ammonia closely follows the lactate response during exercise [4], it lends itself to monitoring muscle fatigue.

##### Hypoxanthine and xanthine (oxipurines)

Hypoxanthine is a naturally occurring purine derivative, which occasionally is a constituent of nucleic acids, where it is present in the anticodon of tRNAs in the form of inosine. Hypoxanthine and xanthine are derived from the degradation of purinic nucleotides (adenine, guanine) [33]. Hypoxanthine is produced by the action of xanthine oxidase on xanthine. More frequently, however, hypoxanthine is formed by xanthine oxidoreductase from the reduction of xanthine. Hypoxanthine-guanine phosphoribosyltransferase converts hypoxanthine into IMP in nucleotide salvage. IMP is degraded to inosine and hypoxanthine by a 5’-nucleotidase [33]. Hypoxanthine and xanthine are usually analysed in the serum or urine.

Reference limits for hypoxanthine were reported to range between 0 and 8 μmol/l [34]. Influences on the variability of these biomarkers are largely unknown. Age-dependency of serum hypoxanthine is not well investigated but differences between the genders seem to exist [35]. No reliable data about the dependency of hypoxanthine on the physical fitness are available. During and after prolonged isokinetic exercise to exhaustion, performed by healthy males in a concentric mode, serum hypoxanthine and xanthine levels were found to significantly increase immediately after exercise depending on the amount of joint excursion [33], rendering hypoxanthine useful for monitoring the metabolic stress of muscle tissue during training or rehabilitation programs [33]. Since serum hypoxanthine is directly correlated with the amount of ATP consumed inside the cell [33], it is a good biomarker of muscle fatigue [36].

#### Oxidative stress biomarkers

One of the most promising groups of biomarkers of muscle fatigue is that related to oxidative stress, which is characterized by the enhanced production of ROS (free radicals) [37]. ROS have an electron in excess, which not only cause fatigue but also mitochondrial damage. Increased ROS production during exercise results in the oxidation of proteins, lipids or nucleic acids [38]. The production of ROS during fatigue is also accompanied by a marked reduction in the antioxidant capacity, which is partially proportional to the training load [10]. ROS are capable of activating the transcription factors known to regulate IL-6 [9]. The production of ROS during exercise is occasionally accompanied by inflammation or soreness, and increases in a biphasic manner [39]. Though influence of ROS on muscle fatigue is complex and elevation of a marker after exercise does not necessarily indicate that it is a key determinant of fatigue, measuring oxidative damage biomarkers, such as lipid peroxidation biomarkers, (thiobarbituric acid-reactive substances (TBARS), Isoprostanes) protein oxidation biomarkers (protein carbonyls) or biomarkers of antioxidant capacity, (glutathione (GSH), glutathione peroxidase (GPX), catalase, total antioxidant capacity (TAC)) appears to be promising to assess fatigue.

### Thiobarbituric acid-reactive substances (TBARS)

Thiobarbituric acid-reactive substances (TBARS) are low-molecular weight end products formed during the decomposition of lipid peroxidation products that react with thiobarbituric acid to form a fluorescent red adduct. TBARS occur in the serum most likely due to peroxidation of low-density lipoproteins and oxygen-mediated injury of myocyte membranes [40]. TBARS are indicators of lipid peroxidation and oxidative stress [41]. TBARS are usually determined in the serum but are also detectable in the saliva [42].

Reference limits for serum TBARS at baseline were reported as 6.8-8.0 μM [10] but are influenced by a number of variables. TBARS increased with age in erythrocytes [43] and were reported to be lower in females compared to males [44]. TBARS increased with increasing physical fitness [10]. Serum concentration of TBARS increased immediately after exercise and again after a delay of several hours. Although exercise-induced oxidative stress is highest immediately after exercise, lipid peroxidation can take place even some time after exercise [45]. In healthy subjects, TBARS increased 5 min after an incremental cycling exercise and during static exercise [23,46]. In healthy males undergoing seven multi-joint resistance exercises six times a week with 1-6 repetitions at 85-100% of maximal strength (overtraining), a >48-h delayed increase of TBARS by 56% compared to baseline was observed [10]. This increase was triggered by leukocyte and macrophage infiltration or xanthine oxidase activation and attributed to the ischaemic reperfusion process. In healthy males a period of intensified training elicited an increase of lipid peroxidation in 96% of the cases, but only after high-volume and very-high-volume training [10]. In patients with CFS, there was a linear correlation between resting fatigue, as assessed by a visual analogue scale, and TBARS [41]. A linear correlation was also found between fatigue and the lag phase, defined as the time necessary to initiate peroxidation, which precedes the formation of conjugated dienes [41]. In CFS patients undergoing incremental exercise to exhaustion, a post-exercise increase in TBARS and a decrease in reduced ascorbic acid were observed [47]. The increase in TBARS was accompanied by delayed but markedly reduced heat shock protein (HSP) 27 and HSP70 levels [47] suggesting that prolonged and accentuated oxidative stress during exercise might result from delayed or insufficient HSP production [47].

#### Isoprostanes

Isoprostanes are prostaglandin-like compounds that, *in vivo*, derive from peroxidation of essential fatty acids (mainly arachnoidic acid) catalysed by ROS and without the action of cyclo-oxygenase. Isoprostanes are esterified to phospholipids in cell membranes and are released in the free form to circulate in body fluids by the action of phospholipases [10]. Isoprostanes are accurate markers of lipid peroxidation and their exercise-associated increase reflects the oxidative damage to cell membranes following muscle activity [10]. Isoprostanes can also act as direct inflammatory mediators and augment the perception of pain. Isoprostanes are usually determined in the serum, urine, or other body fluids and blood cells.

Reference limits of isoprostanes in the serum were reported as 1.5-1.8 ng/ml [10] but depend on a number of influences. Isoprostanes increase with age [48] and have been reported to be slightly lower in females compared to men [49]. Isoprostanes increase with physical fitness [10]. In healthy males, isoprostanes increased 2.4-fold in the urine after low volume training, 4-fold after high-volume training, and 7-fold after very-high-volume training [10]. The increase in isoprostanes was correlated with a decrease in performance and an increase in training volume [10]. In a study of ultra-marathon runners, peripheral F2-isoprostane increased by 57% [50].

#### Protein carbonyls

Protein carbonyls are mainly derived from the oxidation of albumin or other serum proteins [51]. Protein carbonyls are regarded as a marker of oxidative protein injury [52]. Accumulation of protein carbonyls over time is an indicator of oxidative damage to proteins during ageing [53]. Protein carbonyls are usually determined in the serum. Reference limits of protein carbonyls were reported as 0.30-0.36 nmol/mg [10] but vary with a number of conditions [54]. Protein carbonyls increased with age in one study [53] and decreased with age in another [55]. Protein carbonyls were higher in males as compared to females with hypothyroidism [54] and increased with physical fitness [10]. In healthy males, protein carbonyls increased by 50% under intense, high-volume training and by 73% under very-high-volume training [10]. Protein carbonyls remained elevated 96 h post-exercise. In healthy cross-trained men undergoing continuous cycling at 70% of VO_2_max for 30 min and intermittent dumbbell squatting, protein carbonyls increased 1.6-2.4-fold 24 h post-exercise [38]. After ultra-marathon running, elevated protein carbonyls reached the maximum level 48 h post-competition [56]. The post-exercise rise in protein carbonyls is attributed to phagocytic cell invasion into the muscle, which generates ROS and is accompanied by inflammation and soreness [57]. In overtrained athletes protein carbonyls increased already before exercise.

#### Glutathione (GSH)

Glutathione (GSH) is a pseudo-tripeptide that is present in nearly all cells at high concentrations. Glutathione is one of the most important antioxidants and an important reserve of cysteine. GSH is also important for the phase-II biotransformation of toxic substances. For this purpose, halogen-, sulphate-, sulphonate-, phosphate- or nitrate groups are substituted by GSH. In addition to ROS scavenging, GSH is required for the regeneration of ascorbic acid and α-tocopherol [58]. GSH is not only present in cells but is detectable also in the serum and saliva [42].

Reference limits of GSH in the serum were reported as 0.36-0.41 mM but depend on a number of conditions [10]. GSH levels were lower in males compared to females and decreased with age [59] and with increasing physical fitness [10]. GSH in erythrocytes decreased by 31% after high-volume training in healthy trained males [10]. Simultaneously, oxidized glutathione (GSSG) increased by 25% and the GSH: GSSG ratio, another valid oxidative stress marker, decreased by 56% [10]. The decrease in the GSH: GSSG ratio was highly correlated with a drop in performance and a training volume increase, suggesting that the supply of GSH is not sufficient to match its enhanced utilization during overtraining or that the clearance of GSH from the blood is increased [10]. A decrease in GSH was also observed during static exercise [37]. Under continuous cycling at 70% of VO_2_max for 30 min and intermittent dumbbell squatting, GSH decreased by 21% whereas GSSG increased by 25% [38]. In healthy non-smokers the consumption of GSH was maximal 5 min after an incremental cycling exercise [37].

#### Glutathione peroxidase (GPX)

Glutathione peroxidase (GPX) is an enzyme that scavenges hydrogen peroxide (H_2_O_2_) at low levels of exercise, while at higher training volumes the production of H_2_O_2_ exceeds the capabilities of GPX. To compensate for the insufficient clearance of H_2_O_2_ by GPX, the production of catalase is increased at higher levels of exercise [10]. H_2_O_2_ is formed by dismutation of superoxide (O_2_-), which is formed by xanthine-oxidase during the degradation of IMP to uric acid [60]. Excess of H_2_O_2_ causes loss of muscle contractility and thus augments muscle fatigue [60]. GPX occurs ubiquitously and can be found in most body fluids and cells.

Reference limits of GPX in peripheral blood mononuclear cells were reported as 509.3 ± 26.3 U/g protein in 16 healthy controls [61] but reference values depend on various factors. GPX activity in erythrocytes decreased with age [62]. GPX was sex-dependent and higher in females under chronic stress as compared to males [63]. GPX-activity in erythrocytes was also dependent on the physical fitness since it increased in sedentary females performing Tai Chi during 8 weeks [64]. In healthy subjects, GPX increased with the intensity of exercise [10,65]. GPX decreased by 50% in neutrophils and increased by 87% in lymphocytes [66]. In this study GPX increased already below peak oxidative stress levels.

#### Catalase

Catalase is an ubiquitously occurring enzyme that catalyses the decomposition of H_2_O_2_ to water and oxygen. The enzyme has one of the highest turnover rates, converting millions of H_2_O_2_ molecules per single catalase molecule each second. The enzyme is a tetramer with polypeptide chains that are more than 500 amino acids long. Catalase is usually determined in the serum [10].

Reference limits of catalase are reported to range from 96.8 to 129.8 MU/1 in 1756 healthy individuals but depend on various influences [67]. In this study catalase values were lower in females and decreased with age [67]. On the contrary, no gender difference was reported in healthy sport students [68]. In the latter study, catalase increased after short-distance running [68]. Catalase levels do not seem to be dependent on the physical fitness of the probands [69]. In healthy males, serum catalase levels increased only after very-high-volume training [10]. A positive correlation between catalase levels and training volume was also reported in other studies [65]. In professional cyclists, the catalase levels in neutrophils decreased by 40% 3 h post-competition [66].

#### Total antioxidant capacity (TAC)

TAC is defined as the sum of antioxidant activities of the nonspecific pool of antioxidants, consisting of antioxidant enzymes (GPX, catalase, superoxide dismutase), metal chelators, and nonspecific antioxidants (GSH, ascorbic acid, albumin, uric acid, tocopheroles, carotenoids, coenzyme-Q, bilirubin, and amino acids (cystein, methionine, tyrosine)). The non-enzymatic antioxidant properties of the TAC are usually measured by means of the ferric reducing antioxidant power (FRAP) reagens [70]. TAC is usually measured in the serum [10].

Among 13 healthy subjects, aged 18-22y, TAC ranged between 1.26 and 1.89 mmol/l [71]. TAC seems to increase with age since protein carbonyls and advanced oxidation protein products (AOPPs) increased with age and were correlated with TAC [72]. In a Chinese cohort, TAC levels were higher in males as compared to females [73]. No data about the dependency of TAC on the physical fitness were available. In a cohort of sedentary healthy males, TAC decreased after treadmill exercise [71]. In healthy males a period of intensified training elicited a biphasic TAC response, a significant increase after low- and high-volume training, and a decline after very-high-volume training [10]. After all training stages uric acid increased and accounted for about one third of the TAC increase [10]. Additionally, GSH contributed to the increase in TAC [10]. Uric acid and urea also increased immediately after a soccer game in female players and returned to baseline no earlier than 21 h later [51]. The increase in TAC suggests that the body’s antioxidant defense system is activated during exercise [74]. Mobilization of antioxidant tissue stores may help to maintain the antioxidant status if needed [75]. When measuring the non-enzymatic antioxidant properties after exercise, TAC decreased in healthy controls taking a placebo and in patients being treated with a cysteine donor [70]. In patients with a mitochondrial disorder, TAC decreased after a series of 3 minute incremental exercises (60-70 revolutions/min) [70]. In this study, work was increased from 25 W to 70% of the predicted maximal power output or until the highest workload that could be maintained [70].

#### Inflammatory biomarkers

In addition to ATP-depletion and ROS production, exercise and fatigue trigger an inflammatory reaction. After exercise, T-lymphocytes are mobilized into the blood. Available data indicate that contracting skeletal muscle releases myokines (cytokines produced from muscle) to create a systemic anti-inflammatory environment against this inflammatory reaction and to exert specific endocrine effects on visceral fat [8]. This anti-inflammatory reaction has recently been described by Brandt and Pedersen as the myokine concept, which regards the skeletal muscle as an endocrine organ [8]. The concept relies on the fact that muscle fibres produce, express, and release cytokines and other peptides that exert paracrine or endocrine effects [8]. If these endocrine or paracrine functions are not regularly stimulated by muscle contractions, dysfunction of the muscle (muscular endocrine dysfunction) and other organs and tissues may follow, resulting in cardiovascular disease, malignancy or dementia [8]. Myokines produced by the skeletal muscle include IL-6, IL-8, IL-15, brain-derived neurotrophic factor, leukaemia inhibitory factor, fibroblast growth factor 21 and follistatin-like-1 [8]. Myofibrils also release mechano-growth factors, which impart autocrine effects on the muscle fibre. Relevant inflammatory biomarkers are catabolic biomarkers [18] and include cells involved in the cellular immune response, pro-inflammatory cytokines, such as ILs, IL-receptor antagonists (IL-RA), TNF-α [76] and other markers of inflammation [18].

##### Leukocytes

Aerobic physical activity elicits the mobilization of T-lymphocytes into the blood from peripheral lymphoid compartments immediately after exercise, which is quickly followed by lymphocytopenia during the recovery phase [77]. This effect is most pronounced for cluster of differentiation (CD) 8 and natural killer cell populations [77]. Recruitment of T-lymphocytes from the marginal pool represents a nonspecific immune response that occurs in the presence of ischemia of a stressed tissue but in the absence of a real injury [33]. Since immune cells generate ROS, the simultaneous promotion of post-exercise inflammation, tissue removal and healing occur [10].

Reference limits of T-lymphocytes depend on age, sex, and the method applied [78]. T-cells expressing CD8 were reported to be lower in old as compared to young healthy subjects [79] but other studies showed T-lymphocyte subpopulations to increase with age [78]. Sex differences in the T-cell immune responses are particularly evident in diseased subjects [80]. In healthy subjects of any age, CD4+ and CD8+ lymphocytes are mobilized into the peripheral blood compartment after exercise [77]. Also, CD3+, CD4+, CD8 bright and CD56+ lymphocytes were found to increase immediately after an intensive treadmill running protocol in aerobically trained male runners [8]. In this study, the percentage of CD3 + -T-lymphocytes expressing killer cell lectin-like receptor subfamily G1 (KLRG1) and CD57 increased with exercise. After 1 h of recovery, mobilized T-lymphocytes expressing KLRG1 and CD57 disappeared from the peripheral blood compartment [8]. An increase in CCR5+, CCR5+/CD8 and CCR5+/CD45RO-T-cell subpopulations was also reported in frail patients [81]. These changes indicate that T-cells mobilized by exercise have an advanced stage of biological aging and a reduced capacity for clonal expansion compared to T-cells resident in the blood [8]. In addition to lymphocytes, also neutrophils showed a significant increase immediately after exercise in healthy males during prolonged, isokinetic exercise, performed in a concentric mode at different joint excursions [33].

##### Interleukin-6

Interleukin-6 (IL-6) belongs to a group of cytokines that regulate the body’s inflammatory reaction. IL-6 acts as both a pro-inflammatory (monocytes, macrophages) and anti-inflammatory (myocytes) cytokine [8,82]. IL-6 is secreted by T-cells and macrophages in order to stimulate the immune response to trauma or other tissue damage, leading to inflammation. IL-6 cleaves the receptor-subunit glycoprotein gp130. Additionally, IL-6 functions as a myokine, which increases in response to muscle contractions [8,9,83]. Serum IL-6 exponentially increases with exercise duration up to 100-fold, is rapidly released into circulation, precedes the appearance of other cytokines, peaks immediately post-exercise, and returns to resting levels within a few hours of recovery [83,84]. The exponential increase is related to exercise duration, intensity, the muscles engaged in mechanical work and endurance capacity [83]. During exercise, IL-6 is thought to act in a hormone-like manner to mobilize extracellular substrates or augment substrate delivery [85]. IL-6 is markedly produced during the post-exercise period when insulin action is enhanced, but IL-6 is also associated with obesity and reduced insulin action [8]. IL-6 increases glucose uptake, hepatic glucose production during exercise, insulin-mediated glucose disposal, lipolysis and fat oxidation via activation of AMPK or PI3-kinase [8,9]. IL-6 is secreted by osteoblasts to stimulate osteoclast formation and by smooth muscle cells in the tunica media of blood vessels as a pro-inflammatory cytokine. Its role as an anti-inflammatory cytokine is mediated through its inhibitory effects on TNF-α and IL-1, and the activation of IL-1RA and IL-10 [8,83]. IL-6 is usually determined in the serum and probably the most frequently studied cytokine.

Among 54 healthy controls, reference limits of IL-6 ranged between 1.0 and 4.8 pg/ml but were sex-related [86]. IL-6 serum levels were independent of age but were reported lower in females as compared to controls [79]. IL-6 levels are typically increased in inflammatory and immunological disease [86]. In a study of sedentary subjects and athletes, IL-6 levels increased with isometric force and mechanical fatigue [87]. IL-6 also increased in highly trained endurance athletes in response to four weeks of intense running [88]. However, IL-6 did not increase during incremental exercise to exhaustion in patients with CFS [47]. The increase of IL-6 during exercise may be blunted by the ingestion of carbon-rich food [84] but not by fat. In a study of 716 community-dwelling subjects the decrease in maximal isometric hand-grip strength over 6 years was predicted by high IL-6 and IL-1RA levels [12]. IL-6 can also be determined in the saliva [87], but no significant relationship was found between serum and salivary IL-6 levels under resting or post-exercise conditions [87]. When recombinant IL-6 is actively administered, it impairs the performance of long-distance runners and increases the sensation of fatigue in trained runners [84]. Fatigue resistance was also found to be worse in patients with high levels of IL-6 compared to patients with high IL-6 but low HSP70 levels [89].

##### TNF-α

TNF-α is a pro-inflammatory cytokine that is predominantly produced by macrophages and able to induce apoptosis, inflammation, cell proliferation and cell differentiation, and to inhibit tumourgenesis and viral replication. TNF-α is part of the cytokine system, which modulates the organo-neogenesis. TNF-α indirectly causes insulin resistance by increasing the release of free fatty acids from fat tissue and increasing lipolysis in adipocytes [8]. Dysregulation of TNF-α is implicated in the development of cancer, can induce fiver and contributes to the development of cachexia. It also mediates limb muscle contractile dysfunction through the TNF-receptor type 1. TNF-α can be determined in the serum and various cells.

Reference limits of TNF-α under resting conditions ranged between 0.78 and 3.12 pg/ml [71] and seem to be independent of age, sex, and physical condition. Expression of TNF-α mRNA in fibroblasts increased with age [90] but TNF-α serum levels were not different between young and old controls [79]. Sex differences of TNF-α have not been reported in a study of 28 controls [79]. TNF-α levels did not change with increased physical fitness [71]. In patients with sarcoidosis undergoing a maximal incremental cycling exercise test, muscle fatigue was associated with increased serum levels of TNF-α [3]. In a study of 716 community-dwelling subjects, the decrease in maximal isometric hand-grip strength over several years was associated with increasing TNF-α level [12].

### Genetic response biomarkers (mRNA upregulation)

Exercise results in the upregulation of several genes, as can be demonstrated by increases in mRNA for these genes. For example, the transcriptional rate of the IL-6 gene is markedly enhanced and IL-6 mRNA upregulated during muscle contraction [83]. In resting muscle, the IL-6 gene is silent but is rapidly activated by contraction [83]. Like IL-6, IL-8 mRNA was increased after a 3 h treadmill run and after a 1 h cycle ergometer exercise without a concomitant increase in serum IL-8 levels [9]. In 19 patients with CFS, who were studied during a 25 min cycling exercise to determine the amount of mRNA of metabolite-detecting genes (ASIC3, P2X4, P2X5), adrenergic genes (A2A, B-1, B-2, COMT) and immune system genes (IL-6, IL-10, TNF-α, TLR4, CD14), the activity of the genes encoding for ASIC3, P2X4, P2X5, B-1, B-2, COMT, IL-10, TLR4 and CD14 increased significantly in blood lymphocytes 30 min, 24 h and 48 h post-exercise [91]. The gene activity persistently increased between 0.5-48 h after exercise [91], suggesting that dysregulation of metabolite-detecting receptors occurs during fatigue [76,92]. In a study of subjects exposed to exhaustive exercise, both serum IL-6 and muscle IL-6 mRNA increased [93]. Furthermore, severity of fatigue may be influenced by under- or over-expression of selected genes, which interact with mitochondrial apoptosis and biogenesis.

### Potential biomarkers of muscle fatigue

Potential BPMFs due to oxidative stress include H_2_O_2,_ vitamin E, and the nonspecific antioxidants albumin and ascorbic acid. In patients with CFS, vitamin E levels in the serum significantly decreased with increasing fatigue [10]. Five minutes after an incremental cycling exercise, the maximal consumption of plasma ascorbic acid and a slight decrease in the total antioxidant capacity were reported [37]. Further potential biomarkers of fatigue due to oxidative stress could be HSP27 and HSP70, which protect cells against oxidative stress but decreased after an incremental cycling test in CFS patients. AOPP represent another potential biomarker of oxidative metabolism during muscle fatigue. AOPP derive from the peroxidation of proteins such as oxidized albumin [94]. AOPP have no oxidant properties and correlate with dityrosin and the advanced glycation end product pentosidine, which both represent other indices of oxidant-mediated protein damage. Other potential immunological biomarkers include C-reactive protein, high-sensitivity C-reactive protein, IL-1RA, IL-8, IL-10, IL-15, TNF-αR1, and plasma DNA [8,18,95].

### Parameters unsuitable as biomarkers

A number of biomarkers turned out unsuitable as a biomarker for muscle fatigue during exercise.

Parameters that failed to serve as biomarkers of muscle fatigue so far include elastase, IL-1beta and complement C4a [8,95]. The concentration of these markers did not change substantially after sub-maximal exercise or self-paced limited exercise in CFS patients and controls [96]. Another parameter that does not seem to be a predictor of a decrease in muscle strength is the insulin-like growth factor [18]. In a study of muscle fatigue in patients with sarcoidosis, this parameter did not increase in the patient group [3]. Although brain-derived neurotrophic factor mRNA and protein expression are increased in skeletal muscle after exercise, the muscle-derived and brain-derived neurotrophic factor are not released into the circulation [8].

### Accuracy of biomarkers

Although biomarkers appear to be a valuable tool for measuring and monitoring muscle fatigue, it is still under debate, which ones are reliable and the most relevant for clinical use. Among the many biomarkers reported here, the most accurate and valid biomarkers of muscle fatigue are serum lactate and IL-6, but ammonia, oxipurines, the lymphocyte counts or oxidative stress parameters were also investigated for their impact on muscle fatigue. The best biomarkers for monitoring high-intensity muscle fatigue include lactate, IL-6, TBARS, oxipurines and inorganic phosphate [95]. Biomarkers which best reflect low-intensity fatigue include lactate, leukocytes and TBARS. Recovery from muscle fatigue and the resumption of normal function is best monitored by serum lactate, the leukocyte count and IL-6. An example of the clinical application of lactate determination during muscle fatigue is the lactate stress test. This test is carried out during a constant or incremental workload and indicates disturbed oxidative metabolism. There is, however, no general agreement on the sensitivity or specificity of the lactate stress test. Some authors regard it as being highly useful [96], whereas others consider this test inappropriate [97]. In a study of 291 patients with mitochondrial disorders, the sensitivity/specificity of the lactate stress test was 66/84%, respectively [96]. In a study of 15 patients, however, the sensitivity/specificity of the lactate stress test was only 27/86%, respectively [97], but in this study the workload was incremental and not constant.

## Conclusions

Biomarkers are reliable tools for measuring and monitoring muscle fatigue, but the parameters that are the most applicable for this purpose are still being debated. Reasons for the unsatisfactory situation are that validity and reliability, but also proven effectiveness in assessing muscle fatigue, are strongly dependent on the wide range of settings (at rest in the clinic, before during or after exercise in the laboratory) and on the range of population groups (elite athletes, moderately active individuals, sedentary individuals). Other reasons are the still poorly understood physiological changes that skeletal muscle undergoes during fatigue and the huge number of candidates for biomarkers of muscle fatigue, which have either been barely investigated or do not fulfill the criteria of a biomarker. Furthermore, the causal relationship between the biomarkers described and fatigue still needs to be proven, although some correlate substantially with the loss of muscle function. Different assay methods for the discussed biomarkers may also have an impact on their reliability. So far, the most frequently investigated and most widely applied biomarkers of muscle fatigue are serum lactate and IL-6. Additionally, ammonia, leukocytes and oxidative stress parameters are gaining increasing attention. Biomarkers of muscle fatigue could be a prognostic tool for identifying subjects at increased risk of strength decline, but it is unknown whether the interventional lowering of biomarkers can prevent or alleviate muscle strength loss during exercise.

## Competing interests

There are neither financial nor non-financial competing interests.

## Authors’ contribution

JF was responsible for the design, literature search, discussion and writing of the text.

## Pre-publication history

The pre-publication history for this paper can be accessed here:

http://www.biomedcentral.com/1471-2474/13/218/prepub
